# Oxytocin Neurones: Intrinsic Mechanisms Governing the Regularity of Spiking Activity

**DOI:** 10.1111/jne.12358

**Published:** 2016-04-25

**Authors:** J. Maícas Royo, C. H. Brown, G. Leng, D. J. MacGregor

**Affiliations:** ^1^ Centre for Integrative PhysiologyUniversity of EdinburghEdinburghUK; ^2^ Centre for Neuroendocrinology and Department of PhysiologyUniversity of OtagoOtagoNew Zealand

**Keywords:** electrophysiology, oxytocin, supraoptic nucleus, computational modelling

## Abstract

Oxytocin neurones of the rat supraoptic nucleus are osmoresponsive and, with all other things being equal, they fire at a mean rate that is proportional to the plasma sodium concentration. However, individual spike times are governed by highly stochastic events, namely the random occurrences of excitatory synaptic inputs, the probability of which is increased by increasing extracellular osmotic pressure. Accordingly, interspike intervals (ISIs) are very irregular. In the present study, we show, by statistical analyses of firing patterns in oxytocin neurones, that the mean firing rate as measured in bins of a few seconds is more regular than expected from the variability of ISIs. This is consistent with an intrinsic activity‐dependent negative‐feedback mechanism. To test this, we compared observed neuronal firing patterns with firing patterns generated by a leaky integrate‐and‐fire model neurone, modified to exhibit activity‐dependent mechanisms known to be present in oxytocin neurones. The presence of a prolonged afterhyperpolarisation (AHP) was critical for the ability to mimic the observed regularisation of mean firing rate, although we also had to add a depolarising afterpotential (DAP; sometimes called an afterdepolarisation) to the model to match the observed ISI distributions. We tested this model by comparing its behaviour with the behaviour of oxytocin neurones exposed to apamin, a blocker of the medium AHP. Good fits indicate that the medium AHP actively contributes to the firing patterns of oxytocin neurones during non‐bursting activity, and that oxytocin neurones generally express a DAP, even though this is usually masked by superposition of a larger AHP.

Neurones code information as patterns of action potential (spike) activity. These patterns reflect an interaction between the afferent input activity and their intrinsic membrane properties. Spike activity influences these intrinsic membrane properties, and hence can alter how a neurone responds to its inputs. In addition, the inputs may be modulated by the activity of the neurone, both as a result of retrograde modulation of afferent activity and as a consequence of recurrent neuronal circuits. Such changes occur over different time scales and by different mechanisms and, as a result, different neuronal types process information differently 1.

The rat supraoptic nucleus contains only magnocellular neurosecretory neurones; all of these neurones project to the posterior pituitary gland where they secrete the hormones vasopressin and oxytocin into the systemic circulation. The homogeneity of this nucleus and the ability to relate neuronal behaviour to physiological function has made this an important ‘model system’ in neuroscience, and these neurones have been studied very extensively by electrophysiological approaches *in vivo* and *in vitro* 2, 3, 4, 5.

Oxytocin neurones in the rat supraoptic nucleus discharge under the influence of randomly arriving excitatory and inhibitory post‐synaptic potentials (EPSPs and IPSPs) 3, 6, 7. Each spike is followed by a hyperpolarising afterpotential (HAP; sometimes called a fast afterhyperpolarisation). It appears that the major contributor to the HAP is activation of I_C_ 8, a Ca^2+^‐ and voltage‐dependent K^+^ current carried by a large conductance (BK) channel that can be blocked by charybodotoxin. The HAP makes the neurone relatively inexcitable for 30–50 ms after a spike, and its effects on spike timing can be mimicked in a modified leaky integrate‐and‐fire model of a neurone by assuming that a spike instantaneously raises the spike threshold, and also that this change decays exponentially 9. This simple model can match, very accurately, the distribution of interspike intervals (ISIs) observed in magnocellular oxytocin neurones *in vivo*.

However, the ISI distribution holds no information about spike patterning that results from serial interdependence of ISIs. For oxytocin neurones, any given ISI is almost independent of the length of the preceding ISI, although this is not true for longer trains: there is an inverse relationship between the length of a train of 6–10 ISIs and the length of the next ISI, and this relationship cannot be explained by the HAP, which only lasts for approximately 50 ms 10. However, the HAP is not the only activity‐dependent conductance change that affects neurone excitability. When strongly activated to fire repeated spikes, oxytocin neurones display a deep and prolonged hyperpolarisation called the afterhyperpolarisation (AHP). This is the result of the summation of small, prolonged hyperpolarisations that accompany each spike 11, 12, 13, 14, 15 resulting from activation of Ca^2+^‐activated K^+^ currents. The AHP has at least two components that differ in their duration: a ‘medium AHP’ carried by small conductance (SK) channels, which can be blocked by apamin, and a ‘slow AHP’, carried by intermediate conductance (IK) channels, which can be blocked by muscarine 16.

There is also an activity‐dependent depolarising afterpotential (DAP) that has at least two components: a ‘fast DAP’, carried by Ca^2+^ activated nonspecific cation channels 17, and a ‘slow DAP’. Two different Ca^2+^ activated mechanisms have been proposed for the slow DAP: an additional nonspecific cation channel 18 and the switching off of a hyperpolarising K^+^ leak current 19. The DAP, by raising post‐spike excitability, encourages bursting, and is mostly associated with vasopressin neurones, but is also found in at least some oxytocin neurones 17, 20. The fast DAP and the medium AHP have similar time courses and tend to mask one another in recorded membrane potential. They can be more easily detected when the other is blocked pharmacologically 17.

With spike interval analysis and model fitting, we infer the presence of afterpotentials from activity‐dependent changes in excitability. We use ‘generalised’ AHPs or DAPs with parameters determined by the detected excitability effects, rather than being derived from a specific ionic current. The detected and fitted AHP or DAP may correspond to a specific current, or may represent the compound action of multiple ionic currents. For example, every spike is followed by a relative refractory period that lasts approximately 50 ms; this we term the HAP, although the HAP has been proposed to have at least two components: a BK channel and an A‐type K^+^ channel 8.

The AHP is thought to be important for ‘shaping’ the intense bursts of spike activity that oxytocin neurones display during the milk‐ejection reflex. The milk‐ejection reflex is a dramatic and exceptional event. An oxytocin neurone firing typically at just a few spikes/s will suddenly discharge approximately 100 spikes in 2–3 s, with a peak discharge rate of up to 100 spikes/s achieved within approximately 100 ms of the burst onset 21. These bursts are followed by a longer period of relative quiescence. This post‐burst quiescence is too long to be accounted for by the AHP alone, and we have proposed that it reflects a suppression of afferent input induced by burst‐evoked release of endocannabinoids 21. The AHP itself is likely to be responsible for the ‘shape’ of the milk‐ejection burst and the manner in which it slows down after the peak of excitation 21. An AHP also plays a role in shaping the prolonged bursts in phasic firing vasopressin neurones 22. In the bursts, an initial peak of rapid spiking drops to a sustained plateau, determined by the competing actions of a slow DAP that sustains the burst and the AHP.

In the present study however, we focus on the more subtle effect of the AHP during the more common nonbursting activity observed in oxytocin neurones. We have previously shown that, by adding an AHP to the model, we can account fully for the serial dependence of ISIs in spontaneous activity 10. From this, we can infer that the AHP restrains the firing rate of oxytocin neurones even at low firing rates, although any other consequences for the information processing properties of oxytocin neurones remain unexplored. In addition, at least some oxytocin neurones (approximately 20%) display a fast DAP with a time course intermediate between the HAP and the AHP 17, and the physiological significance of this is also largely unexplored. Here, we show that, when averaged over intervals of 5 s or longer, the spontaneous firing activity of oxytocin neurones in the rat is surprisingly stable. The spike counts are much more regular than expected from the irregularity of firing observed in short intervals, suggesting that the intrinsic membrane properties of oxytocin neurones preserve a memory of past activity by which activity is ‘smoothed out’. Here, we explored whether the AHP accounts for this behaviour, using statistical analyses and computational modelling, as well as by using data from experimental studies in which the medium AHP was blocked pharmacologically using apamin 23. We go on to discuss the possible physiological significance of a mechanism that stabilises the firing rate on a timescale of 5 s and longer.

## Materials and methods

We analysed extracellular recordings of the spike activity of single neurones in the supraoptic nucleus of adult virgin female rats using a large library of recordings made over many years. The selected recordings were from adult rats anaesthetised with urethane (ethyl carbamate, 1.3 g/kg body weight i.p.) in which the supraoptic nucleus and neural stalk were exposed by ventral surgery, and a femoral vein was cannulated for i.v injection of cholecystokinin (CCK) 24, 25, 26, 27. All of the selected neurones had been antidromically identified as projecting to the neural stalk to identify them as magnocellular neurosecretory neurones, and had been further identified as oxytocin neurones by their excitatory response to i.v. injections of CCK. Full details of experimental procedures have been reported previously 27. Further data on the effects of apamin on the firing patterns of supraoptic neurones were obtained from published studies in female rats under urethane anaesthesia, in which apamin was delivered by retrodialysis to the supraoptic nucleus during recordings from single, identified oxytocin neurones 23.

If spikes were generated independently of the previous incidence of spikes, then the spike trains would constitute ‘Poisson’ processes and exhibit certain well‐established statistical features. Spikes are not independent of past activity for any neurone; most obviously, oxytocin neurones possess a prominent HAP that imposes a long relative refractory period after spikes. Nevertheless, we state here what is expected of a Poisson process, aiming to judge how far and in what way, the statistics of spike trains deviate from randomness.

1. (Inter‐event distributions. For a Poisson process, the probability of an event occurring at any particular time is independent of the time of the preceding event. This implies that the inter‐event histogram (the ISI distribution) can be described by a single negative exponential, and that the calculated hazard function (described below) is constant over time since the last spike.
2. (Data that arise as a random process should show invariant statistical characteristics when these data are shuffled randomly.
3. (Index of dispersion. The variance of the event frequency (σ^2^) is equal to the mean of the event frequency (μ). If spike timings are purely random, the ‘index of dispersion’, σ^2^/μ, should therefore equal 1, should be independent of μ in a sample of data where μ varies, and should be independent of bin‐width.
4. (Coefficient of variation (CV). The mean of the inter‐event interval (μ) is equal to the SD (σ), so the ‘coefficient of variation’, σ/μ, should equal 1 if spike timings are random.

After excluding neurones from our library with a spontaneous firing rate too low to meaningfully calculate measures of variability, and neurones without at least 400 s of stationary activity, we selected stationary periods of activity recorded from 76 oxytocin neurones. By ‘stationary activity’, we do not mean wholly regular activity because fluctuations in activity such as periodic bursting are features of activity that are often themselves generated in an activity‐dependent way. Instead, we mean activity that, in the period concerned, shows no clear progressive trend (in the first few minutes of a recording, neurones may either speed up or slow down before reaching a steady firing rate) and no singular abrupt changes (e.g. as sometimes occur in conjunction with a change in spike amplitude that indicates a movement of the neurone relative to the electrode). We imported event data (spike timings resolved to 0.1 ms) from spike2 28 files into excel (Microsoft Corp., Redmond, WA, USA) worksheets and, from these, calculated firing rate in different bin widths (from 0.5 s to 20 s) and calculated the mean index of dispersion as the variance/mean rate for a given bin width. We converted the sequences of spike times into sequences of ISIs, randomly shuffled these using excel, and converted them to a new sequence of event timings, from which we calculated the values for index of dispersion for shuffled data. We constructed ISI distributions (in 5‐ms bins) and calculated the coefficient of variation of ISIs as the SD/mean. We constructed hazard functions from the ISI data in 5‐ms bins as described previously 29 according to the formula (hazard in bin [t, t* *+* *5]) = (number of ISIs in bin [t, t* *+* *5])/(number of ISIs of length > t). A hazard function plots how the excitability of a neurone evolves after a spike has fired and it reflects the superimposed effects of Ca^2+^‐ and voltage‐dependent currents that are triggered by a spike, and the perturbations of afferent input that result from that spike. To measure log interval entropy, we used interlab software 30, 31, 32.

### Model neurones

To model the behaviour of the oxytocin neurones, we used an integrate‐and‐fire based spiking model described previously 10 and as refined further 22 to model vasopressin neurones. The model uses a 1‐ms step size and is implemented using modelling software developed in C++ with the open source wxWidgets graphical interface library 33. Simulations were run for 1000–100 000 s of simulated activity. Briefly, the model simulates the firing response to Poisson randomly timed, exponentially decaying, inputs, representing EPSPs and IPSPs at mean rates I_re_ and I_ri_. I_ri_ is defined as a proportion of I_re_ given by I_ratio_ and all of the results here use I_ratio_ = 1 so that input rate is controlled using just I_re_. We assumed that EPSPs and IPSPs have equal and opposite magnitude (fixed at 2 mV) and a half‐life (λ_syn_) fixed at 3.5 ms. The model variable V_syn_ represents the summed EPSPs and IPSPs.

The other model variables represent a set of spike triggered influences on membrane excitability: the HAP, the DAP and the AHP. Following a spike, the HAP, DAP and AHP variables are incremented by fixed values k_HAP_, k_DAP_ and k_AHP_, and decay exponentially with half‐lives λ_HAP,_ λ_DAP_ and λ_AHP_. By contrast to the classic integrate‐and‐fire model, there is no post‐spike reset of the variables, allowing the DAP and AHP in particular, with their longer half‐lives, to accumulate across multiple spike intervals.

All the model variables are summed with the resting potential (V_rest_, fixed at −56 mV) to generate the membrane potential V:

$$V=V_{\mathrm{rest}}+V_{\mathrm{syn}}-\text{HAP}-\text{AHP}+\text{DAP}$$

When V exceeds the spike threshold (V_thresh_, fixed at −50 mV), the neurone fires a spike and the ISI is recorded. The large magnitude fast decaying HAP simulates the post‐spike refractory period. The DAP and AHP have more subtle but longer lasting effects that are more activity dependent. The DAP is new to the oxytocin model but follows the form of the fast DAP used in the vasopressin spiking model 22.

### Parameter fitting

The model was fitted to recorded data by generating a matching number of spike intervals and adjusting parameters to match the firing rate, ISI distribution and index of dispersion. Initially, spikes were generated using the default parameters in Table 1, and parameters I_re_ and λ_HAP_ were adjusted to match the firing rate and ISI distribution. The AHP parameters were then adjusted to match the index of dispersion range while maintaining the match to the ISI distribution and firing rate. A similar process was used when adding the DAP. The parameter sensitivity of the AHP and DAP half‐lives was tested by attempting to fit with smaller and larger values (λ_AHP_ = 50, 1000 and λ_DAP_ = 50, 600), compensated by adjusting the respective AHP and DAP magnitude parameters, k_AHP_ and k_DAP_. These tests confirmed that values in the range presented in the Tables 2 and 3 are necessary to produce good fits to the data.

**Table 1:** Default Parameters Used for the Oxytocin Neurone Model

Name	Description	Value	Units
I_re_	Excitatory input rate	300	Hz
I_ratio_	Inhibitory input ratio	1	–
e_h_	EPSP amplitude	2	mV
i_h_	IPSP amplitude	−2	mV
λ_syn_	PSP half‐life	3.5	ms
k_HAP_	HAP amplitude per spike	30	mV
λ_HAP_	HAP half life	7.5	ms
k_DAP_	DAP amplitude per spike	0	mV
λ_DAP_	DAP half life	150	ms
k_AHP_	AHP amplitude per spike	0.2	mV
λ_AHP_	AHP half life	350	ms
V_rest_	Resting potential	−56	mV
V_thresh_	Spike threshold potential	−50	mV

Adapted from MacGregor & Leng 17. EPSP, excitatory post‐synaptic potential; IPSP, inhibitory post‐synaptic potential; PSP, post‐synaptic potential; HAP, hyperpolarising afterpotential; DAP, depolarising afterpotential; AHP, afterhyperpolarisation.

**Table 2:** Model Parameters to Match to the Three Different Neurones Shown in Fig. 5

	A1, A2	B1, B2	C1, C2	C3, C4	C5, C6
Mean rate (spikes/s)					
Model neurone	12.90	3.79	7.40	7.30	7.37
Real neurone	12.93	3.73	7.38	7.38	7.38
Parameters					
I_re_	752	255	352	540	470
λ_HAP_	5.4	9.3	4.9	2	4.7
k_AHP_	0.17	0	0	0.46	0.62
λ_AHP_	350	–	–	350	350
k_DAP_	0	0	0	0	0.6
λ_DAP_	–	–	–	–	215

Parameters not given here were fixed for all neurones as in Table 1.

**Table 3:** Parameters of Models Matched to Data From Neurones Exposed to Apamin

Interval	Neurone 1			Neurone 2			Neurone 3			Neurone 4			Neurone 5		
	Bsl	Ap1	Ap2	Bsl	Ap1	Ap2	Bsl	Ap1	Ap2	Bsl	Ap1	Ap2	Bsl	Ap1	Ap2
FR M	7.37	7.40	8.00	3.75	4.24	3.68	2.86	2.73	2.17	6.55	8.01	10.24	6.12	5.24	4.57
FR	7.38	7.46	7.91	3.73	4.28	3.61	2.80	2.46	1.87	6.50	8.01	10.20	6.10	5.25	4.89
I_re_	470	365	350	255	295	245	245	210	190	470	454	414	610	430	315
λ_HAP_	4.7			7.5			6.0			6.0			11.3		
k_AHP_	0.62	0.40	0.30	0.42	0.54	0.36	0.94	0.78	0.73	1.39	1.15	0.93	1.13	0.95	0.77
λ_AHP_	350			350			500			300			495		
k_DAP_	0.6			0.37			1.1			1.53			1.22		
λ_DAP_	215			350			350			200			295		

Three periods of data were matched: baseline (Bsl), apamin 1 (Ap1) and apamin 2 (Ap2). FR, recorded firing rate; FR M, modelled firing rate. Parameters not given here were fixed as in Table 1.

## Results

**Figure 1:**
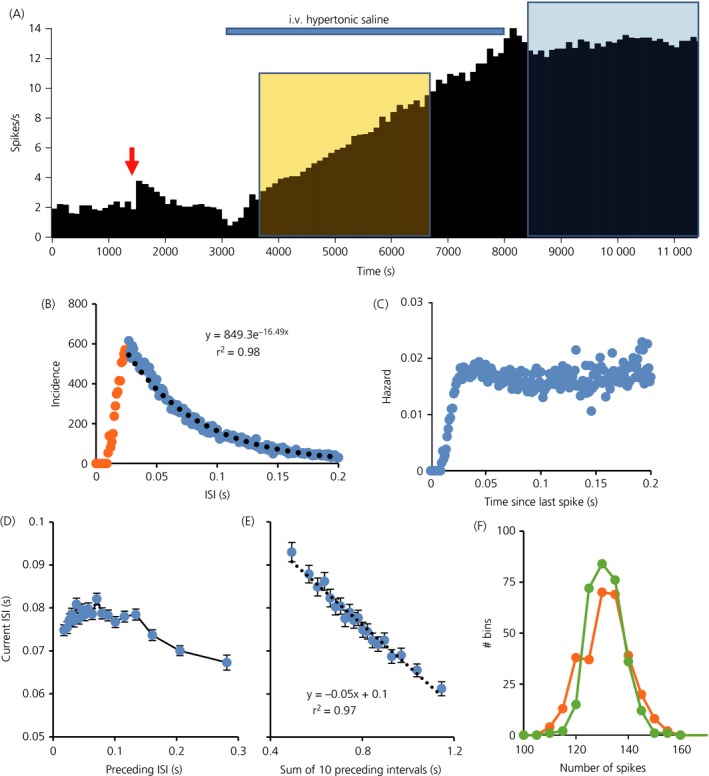
(a) Mean firing rate of an oxytocin neurone (in 100‐s bins) recorded from the supraoptic nucleus of a urethane‐anesthetised rat 27. The neurone was antidromically identified as projecting to the posterior pituitary and identified as an oxytocin neurone by the transient excitation in response to i.v. injection of cholecystokinin (CCK) (red arrow). The neurone was then recorded throughout an i.v infusion of hypertonic saline (blue bar), which increased its firing rate linearly from an initial rate of 2.9 spikes/s to 12.9 spikes/s in this period. (b) Interspike interval (ISI) distribution of this neurone for the 3000‐s period of stable high frequency activity (38798 ISIs) indicated by the blue shaded area in Fig. 1(a). The distribution is typical of oxytocin neurones, displaying a mode at approximately 30 ms and relatively few ISIs shorter than this mode, reflecting a strong post spike relative refractoriness characteristic of a prolonged hyperpolarising afterpotential (HAP). The ISI distribution after the mode (blue symbols) is well fit by a negative exponential (black dotted line, equation of best fit given). This suggests that, after the HAP, spikes arrive apparently randomly. (c) The corresponding hazard function, confirming that the hazard of a spike occurring is independent of the time since the last spike after the end of the period of relative refractoriness. (d) The relationship between each ISI and the preceding ISI, calculated from the same data. Each point plotted is the average of 2000 ISIs, sorted by the length of the preceding ISI. This shows that the length of an ISI is essentially independent of the length of the previous ISI unless the preceding ISI is relatively long (> 150 ms), when there is a weak inverse relationship. By contrast, (e) shows the strong linear relationship between each ISI and the sum of the preceding 10 ISIs. Bars are the SEM (n = 2000). (f) The distribution of spike counts in 10‐s intervals for original data (green symbols) and for randomly shuffled data (orange symbols). The distribution is narrower for the raw data than for the shuffled data.

In initial exploratory analyses, we analysed three long recordings (Fig. 1 a) made in urethane‐anaesthetised male rats in which activity had been slowly increased by i.v. infusions of hypertonic saline (1 or 2 m NaCl at 26–52 μl/min for 30–80 min) 9. As described previously for oxytocin neurones from male rats 29, these neurones displayed ISI distributions that were skewed with very few ISIs shorter than the mode, a long tail that was well fit by a single negative exponential (Fig. 1 b) and a hazard function that rose monotonically over 30–80 ms to a plateau level of excitability (Fig. 1 c). We have previously shown that these ISI distributions closely matched those produced by a leaky integrate‐and‐fire neurone model incorporating an exponentially decaying post‐spike refractoriness, corresponding to a HAP 9. Successive ISIs were relatively independent, although relatively long ISIs (> 150 ms) tended to be followed by ISIs that were shorter than average (Fig. 1 d). However, there was a strong inverse linear relationship between the length of an ISI and the sum of the preceding 10 ISIs (Fig. 1 e). These are typical features of oxytocin neurones that we have previously attributed to the effects of the AHP 10.

**Figure 2:**
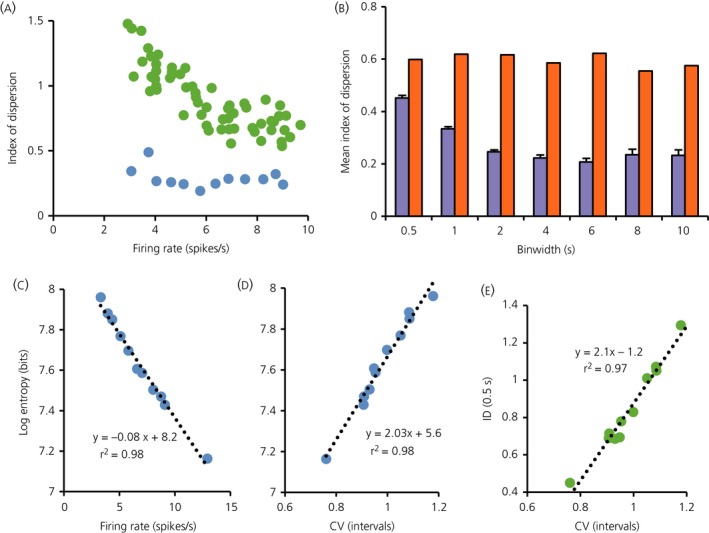
Data are from 3000 s of recording of an oxytocin neurone as its firing rate was increasing linearly in response to an i.v. infusion of 2 m NaCl (orange shaded area shown in Fig. 1). (a) The green symbols are the index of dispersion in 0.5‐s bins measured every 50‐s plotted against the firing rate in that period, and the blue symbols show the index of dispersion in 10‐s bins measured every 250 s. The index is consistently higher for data in 0.5‐s bins. (b) Data from 3000 s of recording of the same neurone firing at a steady rate of 12.9 spikes/s after the end of infusion (blue shaded area in Fig. 1). The index of dispersion was measured every 250 s at different bin widths (0.5,1,2,4,6,8,10 s) and the blue bars show the mean ± SE index of dispersion at each bin width (n = 12). The orange bars show the index of dispersion for shuffled data from this neurone: the ISIs recorded over each 3000‐s period were randomly shuffled, and the index calculated for the shuffled data. The shuffled data still shows an index of dispersion smaller than 1 as a result of the effect of the hyperpolarising afterpotential (which largely prevents very short ISIs) but, at all bin widths, the index of dispersion for the raw data is lower than that for the shuffled data, and it declines with increasing bin width. (c) The relationship, in this neurone, between the log entropy of ISIs (a measure of variability) and firing rate over this period. (d) The relationship between the coefficient of variation (CV; an alternative measure of variability) and log entropy. The two measures show a strong linear correlation with each other and with firing rate, implying that they are essentially equivalent measures and that neither is independent of firing rate. (e) The relationship between CV and the index of dispersion (ID) measured in 0.5‐s bins, also showing a linear relationship.

We examined the regularity of spike activity by looking at the spike rate distribution during periods of stationary activity. For each of the three neurones, the distribution of spike counts in 10‐s bins was symmetrical around the mean, and narrower than the distribution of spike counts for the same set of ISIs after random shuffling to eliminate any order effects (Fig. 1 f): this discrepancy indicates that that the counts in 10‐s bins are more regular than would be expected from the variability of ISIs. We explored this further by calculating the index of dispersion in 0.5‐s bins and in 10‐s bins every 50‐s during the infusion of hypertonic saline that increased the firing rate linearly (Fig. 2 a): at all firing rates, the index of dispersion in 10‐s bins was much lower than in 0.5‐s bins indicating timescale dependent regularity. By contrast, after randomly shuffling ISIs, the index of dispersion of reconstructed spike counts was higher than in the original data, and was independent of bin width (Fig. 2 b).

We studied the relationship between index of dispersion and two other measures of ISI variability: the coefficient of variation and the log interval entropy. For the neurone shown in Fig. 2, the log interval entropy was strongly linearly correlated with firing rate (Fig. 2 c) and with the CV (Fig. 2 d). The CV was also strongly correlated with the index of dispersion as measured in 0.5‐s bins (Fig. 2 e) but relatively weakly correlated with the index of dispersion measured in larger bins (not shown). These relationships held for oxytocin neurones generally; in a sample of 26 oxytocin neurones, log interval entropy was inversely correlated with mean firing rate (r^2^ = 0.46; best fit y = −0.06x + 7.6) and positively correlated with the CV (r^2^ = 0.84; best fit y = 1.8x + 5.9). From this, we concluded that, for oxytocin neurones, the CV and log interval entropy are equivalent measures of interval variability.

**Figure 3:**
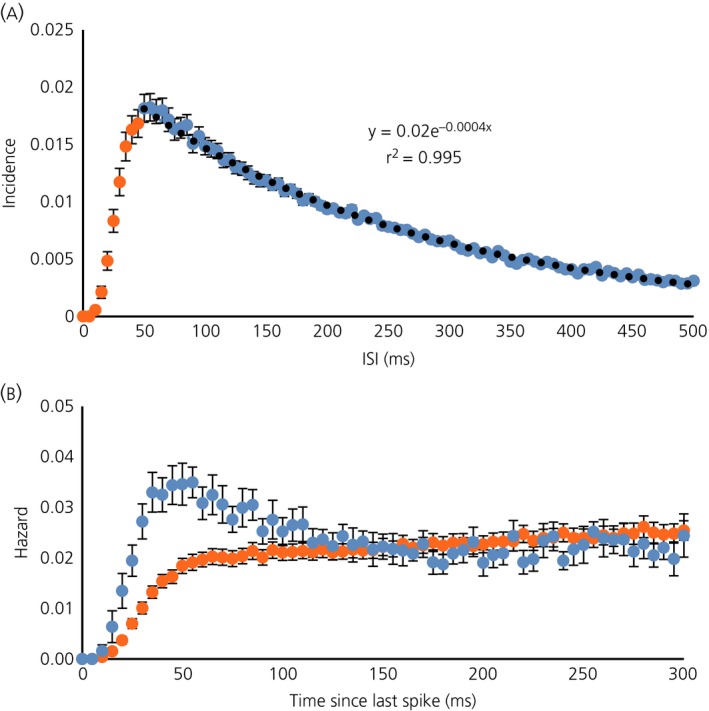
(a) Consensus interspike interval (ISI) distribution for 76 oxytocin neurones from virgin female rats. Histograms were normalised to the total number of ISIs in the period analysed, and the graph plots the mean ± SE incidence in 5‐ms bins. The black dotted line shows a negative exponential fitted to the data from 50 ms onwards (blue symbols). (b) The mean ± SE hazard functions for nine of the 76 neurones that showed a clear post‐spike hyperexcitability (blue symbols) and for the other 67 neurones (orange symbols).

To test the generality of the inferences drawn from this initial exploratory analysis, we analysed 76 oxytocin neurones from virgin female rats 24, 25, 26. These had firing rates of between 1.3 and 8.9 spikes/s (mean ± SD: 3.9 ± 1.8 spikes/s) and the ISI distributions were skewed with modes between 17.5 ms and 112.5 ms (mean ± SD: 61 ± 17) ms). To obtain an ‘average’ distribution, histograms were normalised to the total number of events and averaged. The resulting ‘consensus’ distribution (Fig. 3 a) has a mode at 50 ms and a tail that is also well fitted by a single exponential with a time constant of 250 ms (r^2^ = 0.995 for the fit to ISIs from 50 to 500 ms). The mean hazard function (Fig. 3 b) shows a constant hazard after a post‐spike interval of approximately 50 ms. Thus, for most neurones, the ISI distributions and hazard functions conformed closely to the description that we reported previously for a sample of 23 oxytocin neurones from male rats 29. However, within this larger sample of 76 neurones, heterogeneity was apparent, and nine of the neurones had hazard functions with a conspicuous peak of post spike excitability very like that described previously as typical of magnocellular vasopressin neurones 29, and which apparently reflects a pronounced DAP. These nine neurones had all shown a clear excitation in the 5 min after i.v. injection of CCK (mean change 1.3 ± 0.4 spikes/s) that was similar to the responses of the remainder of the sample (mean change 1.1 ± 0.2 spikes/s).

**Figure 4:**
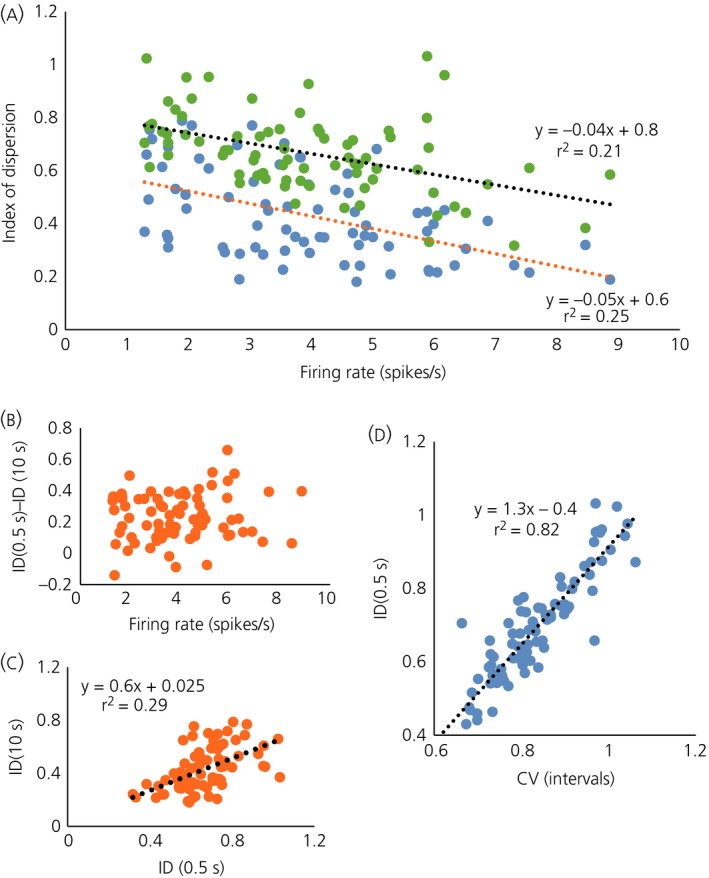
Data from the sample of 76 oxytocin neurones from virgin female rats. (a) The index of dispersion (ID) measured in 0.5‐s bins (blue symbols) and in 10‐s bins (green symbols) plotted against mean firing rate. (b) The difference between the index in 0.5‐s bins [ID(0.5 s)] and that in 10‐s bins [ID(10 s)]. (c) Showing a weak linear correlation between ID(0.5 s) and ID(10 s). (d) Showing a strong linear correlation between ID(0.5 s) and the coefficient of variation (CV).

To calculate the index of dispersion values for the 76 neurones, we analysed 10‐min periods of apparently stationary activity recorded before testing with CCK (Fig. 4) and calculated the values of the index for 0.5‐s and 10‐s bin sizes (Fig. 4 a). At a bin width of 0.5 s, the index of dispersion was strongly correlated with the coefficient of variation of ISIs (Fig. 4 d). Values of the index of dispersion in 10‐s bins were generally lower than in 0.5‐s bins (Fig. 4 b), which is consistent with a greater regularity in 10‐s bins than expected from the regularity observed in 0.5‐s bins. There was only a weak correlation between the index of dispersion in 0.5‐s bins and that in 10‐s bins (Fig. 4 c), suggesting heterogeneity between neurones in the mechanisms underlying this increased regularity.

### Model simulations

We then tested whether model‐generated spike data would show the same characteristics of reduced variability with increasing bin width. The integrate‐and‐fire based model includes mixed random excitatory and inhibitory synaptic inputs and an HAP, and can be extended by adding an AHP and a DAP; the standard parameters that we used are given in Table 1. For each neurone modelled, we attempted to match the mean firing rate, the ISI distribution and the index of dispersion for different bin widths.

**Figure 5:**
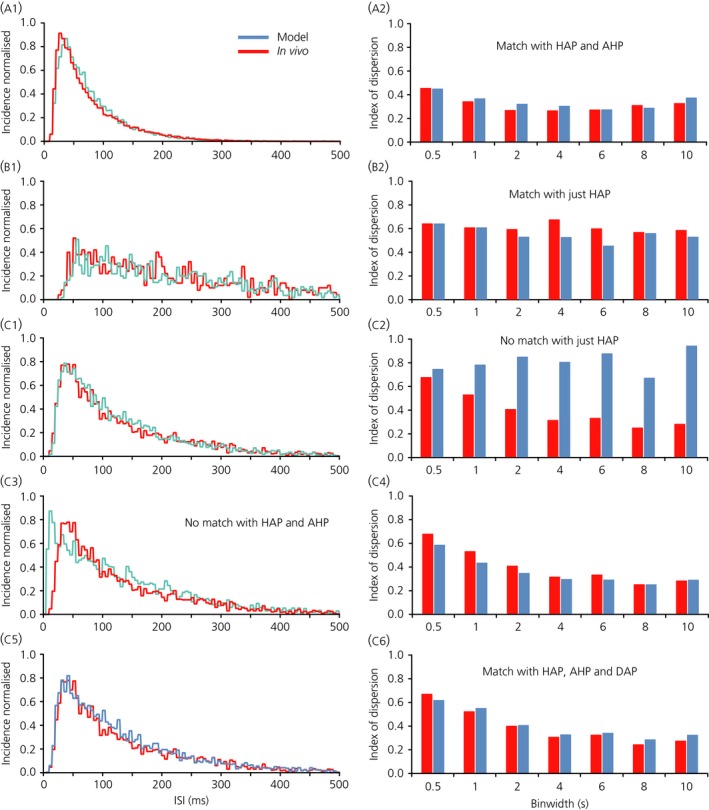
Matching three different neurone baseline intervals. (a) Showing fits for the neurone in Fig. 1 (i.e. for the period of spiking activity indicated by the blue shaded area). A good match was obtained to both the interspike interval (ISI) distribution (a1) and the index of dispersion data (a2) with a model that had a hyperpolarising afterpotential (HAP) and an afterhyperpolarisation (AHP) but no depolarising afterpotential (DAP) (parameters given in Table 2). Note that the index of dispersion is < 0.5 for every bin width. (b) An oxytocin neurone (one of the five subsequently exposed to apamin) where the index of dispersion is similar for every bin width. In this case, it is possible to match both the ISI distribution (b1) and the index of dispersion data (b2) with a model that has just a HAP. (c) Another of the neurones that was tested with apamin. In this case, the index of dispersion decreases as bin width increases (from more than 0.6 for 0.5‐s bins to < 0.3 for 10‐s bins). To obtain a match of both index of dispersion and ISI distributions all three currents (HAP, AHP and DAP) are needed. The model parameters are given in Table 2.

For ‘typical’ oxytocin neurones (i.e. those with hazard functions like the average function shown in Fig. 3 b), parameters could be found for model neurones with no AHP, that gave very close fits to the ISI distributions (Fig. 5 c1). For neurones where the index of dispersion was independent of bin width, it was possible to match both the index of dispersion and the ISI distribution with just a HAP (Fig. 5 b1–b2). When the index of dispersion followed the ‘typical’ decreasing pattern, adding an AHP to the model could result in good fits to the index of dispersion data for all neurones. However, only for some neurones was it possible to simultaneously obtain good fits to the ISI distribution (Fig. 5 a1, a2). Specifically, good fits to both could generally be achieved when the index of dispersion was < 0.6 at a bin width of 0.5 s but, for neurones with an initially high index of dispersion that fell steeply with increasing bin width, we could not obtain good fits to both. For example, for the neurone shown in Fig. 5(c), the ISI distribution could be closely matched by a model with a HAP alone (Fig. 5 c1) but, when we added an AHP to match the index of dispersion data (Fig. 5 c4), we could no longer match the ISI distribution (Fig. 5 c3). For these neurones, the difficulty in simultaneously fitting the index of dispersion data and the ISI distribution arose because an AHP that can account for a low index of dispersion at large bin widths also reduces the index of dispersion at short bin widths. To increase the index of dispersion for low bin widths only, the HAP can be reduced, although this results in an excess of short ISIs in the ISI distribution.

This suggested that we were neglecting another factor increasing variability at short bin widths, and an obvious candidate was the DAP, which tends to amplify high frequency firing. In these neurones, we could fit both the ISI distribution and the index of dispersion data with a model that incorporates a DAP, as well as a HAP and an AHP (Fig. 5 c5–c6).

### Blocking the medium AHP

The model data thus indicated that the index of dispersion data could be explained by the effects of an AHP. However, an AHP sufficiently large to explain the index of dispersion data has, for some neurones, effects that are apparently inconsistent with the shape of the ISI distribution. The modelling suggested that, in addition to the HAP and the AHP, at least some oxytocin neurones may also have a DAP, the overt effects of which may be occluded by the superimposed HAP and AHP.

To test these inferences, we analysed five identified oxytocin neurones that had been exposed to apamin to block one component of the AHP. In these previous experiments 23, neurones had been successively exposed over prolonged periods to two concentrations of apamin administered directly to the supraoptic nucleus by retrodialysis. In each of these neurones, exposure to apamin unmasked a period of post‐spike hyperexcitability consistent with a DAP. Accordingly, we added a DAP to the model, and looked for a fit to the observed distributions before and after each exposure to apamin, and also a fit to the index of dispersion data.

We matched the model to each neurone by finding parameters for the HAP, AHP, DAP and synaptic input rate (I_re_) that closely matched the ISI distribution and the index of dispersion data at baseline (before any exposure to apamin). For the HAP, changing the amplitude has similar effects to changing the half‐life, and so we fixed the amplitude at 30 and varied the half‐life. We thus aimed to find a good match to the three ISI distributions and sets of index of dispersion data for each of the five neurones, with fixed values for the half‐life of the HAP, the half‐life of the AHP and the amplitude and half‐life of the DAP but different values for the amplitude of the AHP and I_re_.

**Figure 6:**
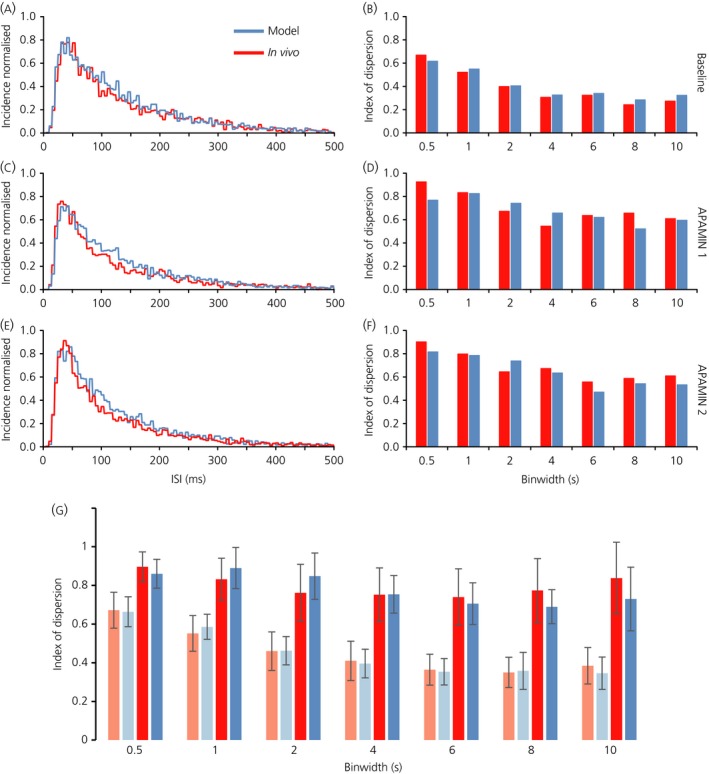
Model fits to five oxytocin neurones that were recorded in basal conditions and during exposure to two concentrations of apamin, given by retrodialysis to the supraoptic nucleus. For each of the five neurones, spike data were fitted by model that included a hyperpolarising afterpotential (HAP), afterhyperpolarisation (AHP) and depolarising afterpotential (DAP); the parameters varied between neurones but, for each of the neurones, we varied only the amplitude of the AHP and the synaptic input frequency to fit the data after apamin**.** The model parameters are given in Table 3. (a–f) Show the fits (in blue) to data (red) from one of these five neurones (neurone 1 in Table 3); the interspike interval (ISI) distributions (a, c and e) are normalised to the total number of events in the period analysed. (g) The mean index of dispersion at different bin widths for the five neurones in basal conditions (light red bars) and after the higher dose of apamin (dark red bars) and the corresponding data from the model neurones (light blue for matches to basal data; dark blue for matches to apamin data).

We found parameter sets that produced good fits for each of the five neurones (Fig. 6 and Table 3). In each case, good fits were obtained to the data after exposure to apamin by reducing both the amplitude of the AHP and the synaptic input rate. The parameter sets that produced good fits are not unique because different combinations of parameters often give equivalent effects but, in every case, including a DAP was essential for fitting the ISI distributions after apamin and, in all but one of the five neurones, including an AHP was essential for fitting the index of dispersion data at baseline. Figure 6(a–f) shows all the fitted data for one of the five neurones under baseline and apamin conditions and Fig. 6(g) shows the mean index of dispersion data for the five neurones and for the five corresponding model neurones.

## Discussion

In these experiments, we have shown that the spiking activity in oxytocin neurones, when measured in bins of 5–10 s, is much more regular than we would expect from the variability of ISIs. This is consistent with a slow activity‐dependent negative‐feedback and likely reflects the actions of the AHP that arises by activation of Ca^2+^‐dependent K^+^ channels. The best fits with the model, with λ_AHP_ in the range 350–500 ms, correspond most closely to the time course of the apamin sensitive SK channel based medium AHP. The half‐life of the AHP determines how quickly it changes and, because it is relatively slow (i.e. taking several seconds to accumulate), its effect on reducing spike interval variability is only detectable in the larger bin sizes. However, a value for *λ*_AHP_ of 1000 ms or greater, which would more closely correspond to the slow AHP, is unable to fit the index of dispersion at the medium bin widths of 2, 4 and 6 s.

However, adding to the model an AHP that is sufficiently large to account for the values of index of dispersion significantly impacts upon the ISI distribution, delaying the mode and impinging on the ability of the model neurones to display high frequency firing, especially as seen during the milk‐ejection reflex. It therefore appears that oxytocin neurones also have an activity‐dependent depolarisation superimposed upon the HAP and AHP, with an intermediate timescale. Together, these three features allow the neurones to maintain a relatively regular firing rate in basal conditions at the same time as retaining the ability to generate bursts of activity.

In fitting models to the data from oxytocin neurones exposed to apamin, we aimed to identify parameters that fitted the behaviour of each of five neurones in three conditions (at baseline and after exposure to two concentrations of apamin). Hazard function based spike interval analysis shows that apamin removes any hazard detectable AHP, and unmasks a distinct DAP, which is enhanced further with an increased dose of apamin 23. For each neurone, we found a parameter set that would fit all three conditions well with changes in just two parameters: the synaptic input rate and the amplitude of the AHP. In each case, the fits involved reducing the AHP amplitude progressively with increasing apamin concentration, consistent with the established actions of apamin to block one component of the AHP, although the fits also required a progressive reduction in synaptic input. This suggests that, in the experimental conditions, apamin also had presynaptic actions, either reducing excitatory input or increasing inhibitory input. The latter is more likely because the actions of apamin would be generally expected to increase neuronal excitability, increasing spike rate as is observed *in vitro* 34, and supraoptic neurones receive an extensive inhibitory input from GABA neurones in the perinuclear zone immediately adjacent to the supraoptic nucleus 35, 36.

Importantly, to obtain these good fits in all conditions, it was necessary in all neurones to include a DAP in the model. The DAP has the opposite effect to the AHP on the index of dispersion; by acting as a positive feedback, it increases spike interval variability at short bin widths. Although vasopressin neurones in the supraoptic nucleus typically display a conspicuous DAP, oxytocin neurones generally do not. However, the presence of a fast DAP has been reported in approximately 20% of oxytocin neurones 17, which is consistent with the observation in the present study of a conspicuous post‐spike hyperexcitability in nine of 76 neurones (12%). In supraoptic neurones generally, DAPs are triggered by Ca^2+^ influx during spikes 13, 37, 38, 39, although their ionic basis is poorly understood. One study suggested that they may result from the Ca^2+^‐dependent reduction of a resting K^+^ conductance 19, although subsequent work suggested that a Ca^2+^‐activated nonselective cation channel is involved 17, 18. The effect of a DAP on oxytocin neurone activity is to increase irregularity of firing, especially when measured in short bin widths; it thus appears that the combination of a DAP and an AHP has the effect of increasing the regularity of firing in long bin widths while protecting the ability to fire at high frequencies during milk‐ejection bursts.

The parameters that we found for the DAP correspond approximately to those reported for the fast DAP 17. A larger subset of oxytocin neurones *in vitro* have been reported to express a slow DAP, with a much longer duration (approximately 2 s) than the fast DAP 3, 20. Because the duration of the slow DAP is similar to that of the slow AHP, it is possible that their effects upon spike excitability largely cancel out in the circumstances that we are exploring them (stable spontaneous activity).

How important it is for an oxytocin neurone to maintain a regular firing rate in constant conditions is hard to judge. For oxytocin neurones generally, slow activity‐dependent mechanisms reduce the index of dispersion in 10‐s bins from approximately 1 to 0.4 at a firing rates of 4 spikes/s. This is equivalent to reducing the SD from 6.3 to 2.8, which is a substantial reduction, although the plasma oxytocin concentration reflects the averaged secretion of several thousand oxytocin neurones. Thus, is this reduction physiologically meaningful? It may well be because, for any one oxytocin neurone, the relationship between firing rate and secretion is complex and nonlinear: for short bursts of spikes, secretion increases disproportionately with spike frequency 40, so that a 1‐s burst at 50 Hz triggers the secretion of approximately 100 times as much oxytocin as is released by the same number of spikes at 1 Hz 41. Moreover, subsets of magnocellular neurones project to sites within the brain, and these sites receive relatively few oxytocin fibres 42. Accordingly, mechanisms that reduce the ‘burstiness’ of firing that arises from random variation in synaptic input may be very important for ensuring that the secretion rate at these sites accurately reflects the mean firing rate.

We can also look more generally at the possible utility of these mechanisms, remembering that subpopulations of magnocellular oxytocin neurones project to many different brain sites and that, at these central projections, they appear to use glutamate as a conventional synaptic neurotransmitter. The mean firing rate of any particular oxytocin neurone is proportional to the plasma sodium concentration, and increases by an average of approximately 0.7 spikes/s for every 1 mm increase. We can therefore ask, if the plasma Na^+^ concentration is raised by 1 mm, for how long do we need to measure the firing rate of an oxytocin neurone to know with 95% confidence that plasma sodium has increased (given no change in any of the other stimuli that influence oxytocin neurones)? Suppose that the starting firing rate ‘a’ is 3 spikes/s, that we know this with certainty, and that the true rate ‘b’ after osmotic stimulation is 3.7 spikes/s. If the index of dispersion is 1, it will be necessary to measure the firing rate of the neurone for at least 30 s to have 95% confidence that the firing rate is actually higher than ‘a’. By contrast, if the index of dispersion is 0.2, then just 8 s is sufficient. In practice, neurones do not have good mechanisms for averaging synaptic inputs over prolonged periods. Thus, if it is important for neuronal networks to respond to small but sustained changes in external signals swiftly and reliably, then cellular mechanisms for reducing the variability of discharge patterning may be very important.
